# Recruitment and baseline data of a multicenter randomized controlled trial for Chinese Hearing Solution for Improvement of Cognition in Elders (CHOICE): Hearing intervention for decreasing risk of developing dementia in elders with mild cognitive impairment

**DOI:** 10.1002/alz70857_096884

**Published:** 2025-12-24

**Authors:** Ying Chen, Lei Guan, Jie Chen, Qiongfei Yu, Hao Wu

**Affiliations:** ^1^ Shanghai Ninth People's Hospital, Shanghai Jiaotong University School of Medicine, Shanghai, Shanghai, Shanghai, China; ^2^ Ear Institute, Shanghai Key Laboratory of Translation Medicine on Ear and Nose Disease, Shanghai Jiaotong University School of Medicine, Shanghai, Shanghai, Shanghai, China; ^3^ Department of Otolaryngology‐Head and Neck Surgery, Shanghai Ninth People's Hospital, Shanghai Jiaotong University School of Medicine, Shanghai, China., Shanghai, Shanghai, China; ^4^ Ear Institute, Shanghai Key Laboratory of Translation Medicine on Ear and Nose Disease, Shanghai Jiaotong University School of Medicine, Shanghai, China., Shanghai, Shanghai, China

## Abstract

**Background:**

Hearing loss (HL) usually indicates high risk of cognitive decline. We intend to investigate whether hearing aids fitting reduces cognitive decline of participants with hearing loss and mild cognitive impairment (MCI) by Chinese Hearing Solution for Improvement of Cognition in Elders‐randomized controlled trial (CHOICE‐RCT), a multicenter, parallel randomized controlled trial.

**Method:**

We screened and enrolled participants aged above 60 years with moderate to severe sensorineural hearing loss and mild cognitive impairment from CHOICE project, outpatients in subcenters and high‐risk population. After collection and quality control, the baseline data was stored in our platform called CRIP. In addition to baseline analysis, we will use statistical methods to explore correlations within it.

**Result:**

Participant recruitment began from May 2021 to February 2024. We screened 20786 participants. 703 were enrolled finally with 408 from CHOICE Cohort, 230 from outpatients and 65 from high‐risk population. Baseline characteristics: mean age was 74.52±7.15 years, 35.7% female; mean MMSE was 23.48±3.05.

**Conclusion:**

Enrolment of study has met the sample size. Follow‐up data collecting is ongoing as planned, which will be completed at 2026.

